# Application of a modified perfusion strategy via anastomosis of the innominate artery with a 10-mm artificial vascular graft in acute type A aortic dissection

**DOI:** 10.3389/fmed.2025.1703615

**Published:** 2025-12-11

**Authors:** Yun Lu, Zhongxin Zhou, Yang Zhang, Jun Wei, Hao Zhang

**Affiliations:** 1 Department of Cardiac Surgery, Xuzhou Clinical School of Xuzhou Medical University, Xuzhou, China; 2 Department of Cardiothoracic Surgery, Affiliated Hospital of Xuzhou Medical University, Xuzhou, China

**Keywords:** acute type A aortic dissection, innominate artery cannulation, antegrade cerebral perfusion, cerebral protection, perfusion strategy, aortic surgery

## Abstract

**Background:**

Acute type A aortic dissection (ATAAD) necessitates emergent surgery with optimal cerebral protection. Traditional axillary artery cannulation carries risks of brachial plexus injury and inadequate perfusion. This study evaluates a modified technique using a 10-mm vascular graft anastomosed to the innominate artery to improve cerebral and systemic perfusion.

**Methods:**

We retrospectively analyzed data from 94 consecutive ATAAD surgical patients between May 2024 and May 2025. All patients underwent hypothermic circulatory arrest (HCA) combined with antegrade cerebral perfusion (ACP). Perfusion was established via end-to-side anastomosis of a 10-mm straight artificial vascular graft to the innominate artery, through which both systemic arterial perfusion and ACP were conducted.

**Results:**

In-hospital mortality was 14.89%. Mean operative times included cardiopulmonary bypass (171.27 ± 43.65 min) and circulatory arrest (25 min). Complications included stroke (10.64%), tracheostomy (17.02%), and acute kidney injury (23.40%). No upper limb ischemia or vascular injuries occurred.

**Conclusions:**

The modified perfusion strategy utilizing innominate artery anastomosis with an artificial vascular graft is a safe, effective, and technically reliable method. It provides excellent cerebral perfusion, unobstructed surgical visibility, and avoids upper limb ischemic complications, making it worthy of clinical promotion.

## 1 Introduction

Acute type A aortic dissection (ATAAD) represents one of the most lethal cardiovascular emergencies, characterized by complex pathogenesis and insidious clinical presentation that often leads to devastating outcomes. Substantial evidence indicates that the mortality rate of untreated ATAAD increases by 1%−2% per hour during the initial phase of hospitalization, particularly within the first 48 h following symptom onset (1). Despite remarkable advancements in surgical techniques and diagnostic modalities over recent decades, operative mortality remains formidable, underscoring the critical importance of refining perioperative management strategies.

In this context, the optimal perfusion strategy during surgical intervention serves as a cornerstone for successful outcomes, directly influencing both cerebral protection and systemic organ perfusion. Contemporary research has established that hemodynamic stability, governed by an appropriate perfusion approach, significantly reduces intraoperative mortality risk and mitigates postoperative complications (2). Conventional perfusion methods, including axillary and femoral artery cannulation, present several limitations that may compromise patient outcomes. These approaches have been associated with substantial risks of cerebral hypoperfusion, embolic events, limb ischemia, and renal insufficiency, all of which profoundly impact postoperative recovery and long-term prognosis (3).

The pursuit of more physiological and effective perfusion strategies has therefore emerged as a surgical priority, particularly for critically ill patients who require rapid establishment of cardiopulmonary bypass to prevent irreversible end-organ damage. Recent technical innovations have focused on developing perfusion methods that not only minimize complications but also reduce crucial intervention time (1). The introduction of innominate artery cannulation via prosthetic graft anastomosis represents one such advancement, offering the potential for more rapid bypass establishment, improved cerebral perfusion dynamics, and reduced procedure-related morbidity.

This study describes and evaluates a modified perfusion strategy utilizing a 10-mm artificial vascular graft anastomosed to the innominate artery, with particular focus on its ability to address the limitations of conventional approaches. We specifically examine how this technique facilitates earlier circulatory support implementation, reduces neurological complications, and optimizes surgical workflow advantages that may be particularly impactful for high-risk patients presenting in critical condition.

## 2 Methods

### 2.1 Study design and ethical approval

This was a single-center, retrospective, observational study. Consecutive patients who underwent emergency surgery for ATAAD in the Department of Cardiac Surgery of our hospital between May 2024 and May 2025 were enrolled. This study was approved by the hospital's Institutional Review Board. Informed consent was waived due to the retrospective nature of the study.

### 2.2 Patient population and selection criteria

#### 2.2.1 Inclusion criteria

a. Age ≥18 years;
b. Preoperative diagnosis of ATAAD (time from onset < 14 days) confirmed by computed tomography angiography (CTA);
c. Underwent emergency surgery utilizing the modified innominate artery cannulation perfusion strategy described in this study.

#### 2.2.2 Exclusion criteria

a. History of severe neurological diseases (e.g., significant residual dysfunction after stroke);
b. Concurrent advanced malignant tumors or other severe underlying diseases with a life expectancy of < 1 year;
c. Traumatic aortic dissection;
d. Dissection extending into the innominate artery;
e. Incomplete clinical data.

A total of 94 patients were ultimately included in the analysis.

### 2.3 Surgical technique in detail

All operations were performed by the same team of surgeons highly experienced in aortic surgery. Procedures were conducted under general anesthesia with standard monitoring, including invasive arterial blood pressure (typically right radial and femoral arteries), central venous pressure, transesophageal echocardiography (TEE), and cerebral oxygen saturation monitoring (near-infrared spectroscopy, NIRS).

### 2.4 Surgical approach and exposure

A median sternotomy incision was performed. The pericardium was longitudinally incised and suspended. The heart and the major branches of the aortic arch were fully exposed. The proximal segment of the innominate artery was carefully dissected free for approximately 3–4 cm.

### 2.5 Core steps of the modified perfusion technique (innominate artery cannulation)

Systemic heparinization: heparin was administered at a dose of 3–4 mg/kg body weight to maintain an activated clotting time (ACT) >480 s (4).

Application of side-biting clamps and arteriotomy: two vascular clamps were applied to completely occlude the proximal and distal innominate artery. A longitudinal incision, approximately 10–12 mm in length (matching the diameter of the artificial graft), was made on the anterior wall of the clamped mid-segment of the innominate artery using a No. 11 scalpel blade. The incision was precisely extended with fine scissors to create a smooth, neat arterial window.

End-to-side anastomosis of artificial graft to innominate artery: a 10-mm × 10-cm straight artificial vascular graft was used. A continuous exerting suture technique was employed (stitch interval approximately 1 mm, edge distance approximately 1.5 mm), maintaining even tension. The suture line was tightened and tied, aiming to create a hemodynamically favorable anastomosis. This step took approximately 5 on average.

**Figure 1:**
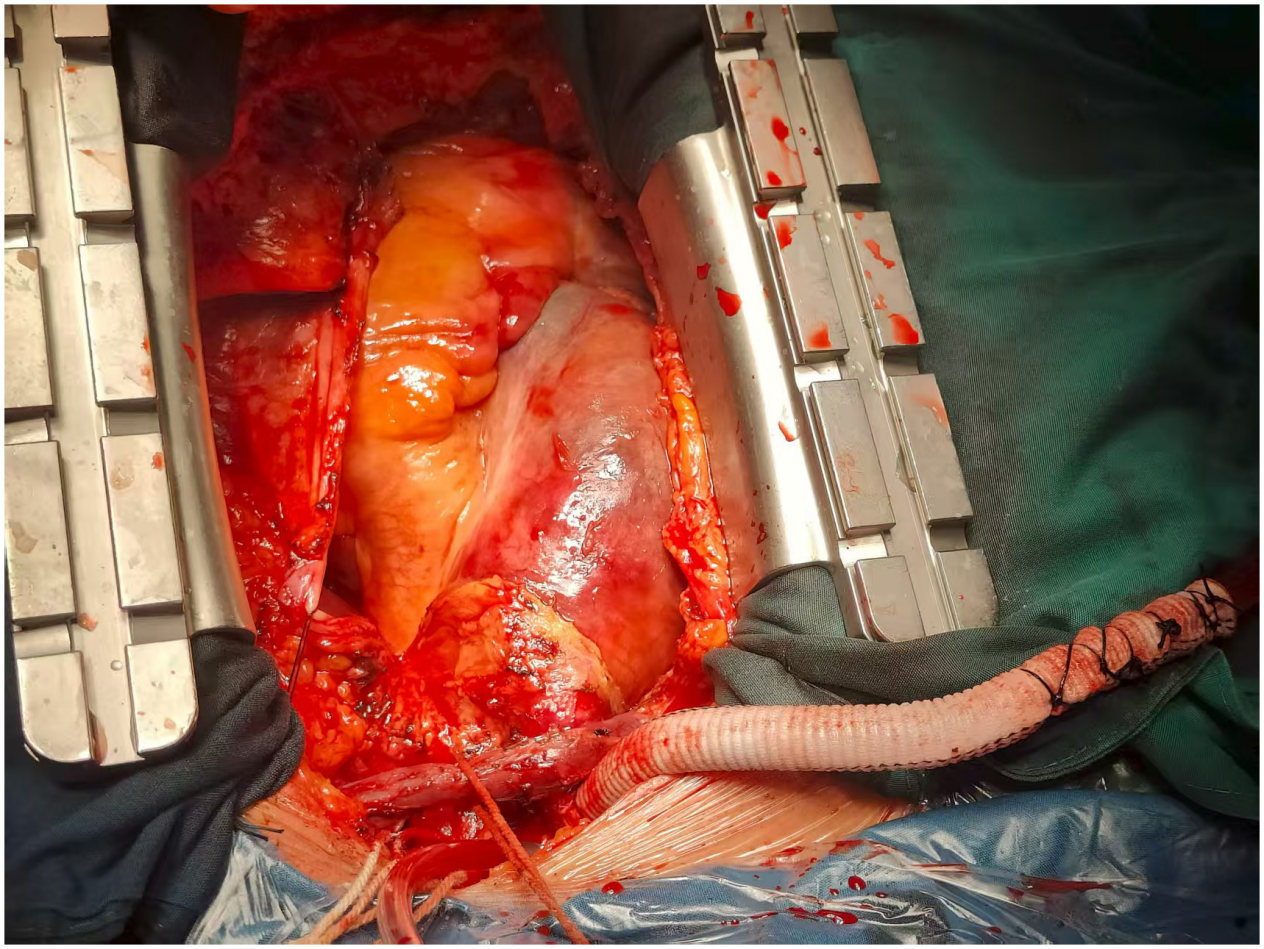
Illustration of innominate artery cannulation using an artificial graft.

Post-anastomosis inspection and cardiopulmonary bypass establishment: the side-biting clamps were slowly and gently released to check for active bleeding at the anastomotic site, which was repaired with additional sutures if necessary. An arterial cannula was inserted into the artificial graft and secured with two layers of #10 silk sutures. The perfusion line was connected to the cardiopulmonary bypass (CPB) machine. Venous drainage was established via cannulation of the right atrium or the superior and inferior vena cava. Figure 1 intraoperative view of the arterial cannulation setup. A 10-mm gelatin-coated polyester graft is anastomosed end-to-side to the innominate artery (IA). The arterial cannula (C) is inserted into the graft and secured with ligatures. The perfusion line is connected to the cannula, establishing antegrade systemic and cerebral perfusion.

### 2.6 Cerebral protection strategy and core surgical procedures

All patients received cerebral protection using moderate hypothermic circulatory arrest (HCA) combined with unilateral antegrade cerebral perfusion (ACP).

When the nasopharyngeal temperature reached 24–28 °C, the root of the innominate artery was clamped, and ACP was initiated through the artificial graft. The ACP flow rate was maintained at 5–10 ml/kg/min, regulated based on monitoring of the right radial artery pressure (maintained at 40–70 mmHg) and cerebral oxygen saturation (5).

During the HCA period, procedures such as aortic root management (e.g., Bentall, Wheat, David procedures), hemiarch, or total arch replacement were performed based on the extent of the dissection. The frozen elephant trunk (FET) technique was widely used in total arch replacement procedures (6).

After completing the arch procedures, systemic perfusion was gradually resumed, rewarming was initiated, and subsequent steps such as proximal anastomosis were completed.

### 2.7 Data collection

Data were collected in three categories.

Preoperative data: demographic characteristics (age, gender), past medical history (hypertension, diabetes, Marfan syndrome, etc.), clinical presentation, and preoperative imaging findings.

Intraoperative data: surgical procedure, cardiopulmonary bypass (CPB) time, aortic cross-clamp time, hypothermic circulatory arrest (HCA) time, ACP flow rate and duration, innominate artery anastomosis time, total operative time, intraoperative blood transfusion volume, etc.

Postoperative outcomes: the primary endpoint was in-hospital mortality. Secondary endpoints included: neurological complications (permanent neurological dysfunction/stroke, transient neurological dysfunction), respiratory function (tracheostomy rate, mechanical ventilation duration), renal function (incidence of acute kidney injury and need for renal replacement therapy), re-exploration rate, surgical site infection rate, myocardial infarction, hoarseness (recurrent laryngeal nerve injury), and technique-specific complications (e.g., perfusion-related upper limb ischemia, brachial plexus injury, anastomosis-related bleeding or tearing).

### 2.8 Statistical analysis

All data were analyzed using SPSS software (version 26.0). Continuous data conforming to a normal distribution are presented as mean ± standard deviation (Mean ± SD); non-normally distributed continuous data are presented as median (interquartile range) [M (IQR)]; and categorical data are presented as number (percentage) [*n* (%)]; Descriptive comparisons of key intraoperative time metrics were made with historical data from published literature on conventional axillary artery cannulation techniques.

## 3 Results

### 3.1 Preoperative patient baseline characteristics and clinical features

This study consecutively enrolled 94 patients who underwent emergency surgery for ATAAD. The detailed preoperative baseline characteristics, comorbidities, and preoperative status of all patients are shown in Table 1. The cohort was predominantly male (78.72 %), with a mean age of 57.45 ± 14.58 years and a mean body mass index (BMI) of 24.93 ± 3.26 kg/m^2^.

**Table 1:** Baseline characteristics of the study cohort with aortic dissection.

**Variable names**	**Level**	**Overall**
Sex (%)	Male	74 (78.72)
	Female	20 (21.28)
Smoking history (%)	No	82 (87.23)
	Yes	12 (12.77)
Hypertension (%)	No	26 (27.66)
	Stage 1	44 (46.81)
	Stage 2	2 (2.13)
	Stage 3	22 (23.40)
Type 2 diabetes (%)	No	90 (95.74)
	Yes	4 (4.26)
Cerebral infarction (%)	No	88 (93.62)
	Yes	6 (6.38)
Atrial fibrillation (%)	No	92 (97.87)
	Yes	2 (2.13)
Preoperative coma (%)	No	88 (93.62)
	Yes	6 (6.38)
Cardiac tamponade (%)	No	64 (68.09)
	Yes	30 (31.91)
Cardiogenic shock (%)	No	78 (82.98)
	Yes	16 (17.02)
Sinus of Valsalva involvement (%)	No	50 (53.19)
	Yes	44 (46.81)
Coronary artery involvement (%)	No	88 (93.62)
	RCA	2 (2.13)
	Both	4 (4.26)

### 3.2 Preoperative comorbidities

Hypertension was the most prevalent risk factor. Based on medical history records, 46.81% of patients had a history of hypertension and were receiving medication, 23.40% had hypertension that was controlled with medication, and 27.66% had no history of hypertension. Other common comorbidities included a history of smoking (12.77%), previous cerebral infarction (6.38 %), and type 2 diabetes (4.26%). Atrial fibrillation was relatively uncommon (2.13%).

### 3.3 Preoperative critical status

A considerable proportion of patients were already in a state of hemodynamic instability or end-organ malperfusion upon admission. Among them, 31.91% of patients had cardiac tamponade, 17.02% were in a state of cardiogenic shock, and 6.38% presented with preoperative coma. Additionally, 2.13% of patients had lower limb malperfusion.

The anatomical characteristics of the aortic dissection were assessed based on preoperative CTA images. The results showed that 46.81% of patients had dissection involving the aortic sinuses. Involvement of the innominate artery was relatively low (4.26%). According to the Stanford classification, all patients had type A dissection. An intimal tear (“laminated crack”) was present in 59.57% of patients, and 17.02% had multiple tears. Coronary artery involvement was observed in 6.38% of patients.

### 3.4 Intraoperative data

All surgeries were successfully completed. Key intraoperative time metrics are shown in Table 2. The total operative time was 277.5 ± 63.93, with the innominate artery to artificial graft anastomosis time being only 5.0 ± 1.5. The cardiopulmonary bypass time was 171.27 ± 43.65, and the aortic cross-clamp time was 109.90 ± 29.22. The core cerebral protection phase deep hypothermic circulatory arrest time was controlled at 21.95 ± 7.58, during which antegrade cerebral perfusion was consistently administered through the established perfusion circuit.

**Table 2:** Intraoperative data.

**Variable names**	**Time (min)**
Surgical duration	277.50 ± 63.93
Cardiopulmonary bypass time	171.27 ± 43.65
Aortic cross-clamp time	109.9 ± 29.22
Postoperative ward transfer time	88.51 ± 92
Hypothermic circulatory arrest time	21.95 ± 7.58
ICU length of stay	88.07 ± 108.77

### 3.5 Postoperative outcomes and complications

The primary postoperative outcome measures are shown in Table 3. There were 14 in-hospital deaths, yielding a mortality rate of 14.89%. The rate of permanent neurological dysfunction (stroke) was 10.64% (10/94). Other major complications included: acute kidney injury in 23.40% (22/94), of which 17.02% (16/94) required renal replacement therapy; respiratory failure requiring tracheostomy in 17.02% (16/94); and 6.38% (6/94) of patients required re-exploration for bleeding.

**Table 3:** Postoperative complication rates in aortic dissection.

**Variable names**	**Level**	**Overall**
In-hospital mortality (%)	No	80 (85.11)
	Yes	14 (14.89)
MODS (%)	No	92 (97.87)
	Yes	2 (2.13)
Postoperative cerebral infarction (%)	No	84 (85.11)
	Yes	10 (14.89)
Postoperative atrial fibrillation (%)	No	92 (97.87)
	Yes	2 (2.13)
Postoperative pleural effusion (%)	No	86 (91.49)
	Yes	8 (8.51)
Secondary thoracotomy (%)	No	88 (93.62)
	Yes	6 (6.38)
Hoarseness after surgery (%)	No	90 (95.74)
	Yes	4 (4.26)
Perioperative myocardial infarction (%)	No	92 (97.87)
	Yes	2 (2.13)
Incision infection (%)	No	90 (95.74)
	Yes	4 (4.26)
Postoperative arteriovenous thrombosis (%)	No	88 (93.62)
	Yes	6 (6.38)
Kidney failure (%)	No	72 (76.60)
	Yes	22 (23.40)
Hemodialysis (%)	No	78 (82.98)
	Yes	16 (17.02)
Tracheostomy (%)	No	78 (82.98)
	Yes	16 (17.02)

The incidence of postoperative new-onset atrial fibrillation was 2.13% (2/94). Other surgery-related complications included: postoperative hoarseness in 4.26% (4/94), surgical site infection in 4.26% (4/94), and postoperative arteriovenous thrombosis in 6.38% (6/94). The incidence of multiple organ dysfunction syndrome (MODS) and perioperative myocardial infarction was low, both at 2.13% (2/94).

Most importantly, no patient experienced direct complications related to innominate artery cannulation, such as anastomotic bleeding, tearing, upper limb ischemia, or brachial plexus injury, demonstrating the safety of this technique.

## 4 Discussion

This study is the first to systematically evaluate the application of a technique establishing an extracorporeal circulation perfusion pathway via end-to-side anastomosis between the innominate artery and a 10-mm artificial vascular graft in surgery for acute type A aortic dissection (ATAAD). Based on the analysis of 94 consecutive cases, this innovative technique proves to be not only safe and feasible but also demonstrates significant advantages in terms of cerebral protection efficacy, surgical efficiency, and complication prevention, offering a new technical option for the surgical treatment of this critical condition, ATAAD.

### 4.1 Superior cerebral protection and neurological outcomes

The permanent neurological dysfunction (stroke) rate in this study was 10.64%, which compared favorably to the 11% stroke rate associated with traditional axillary artery cannulation reported in the International Registry of Aortic Dissection (IRAD) (7). More importantly, given that 6.38% of patients in this cohort had preoperative coma and 17.02% were in cardiogenic shock, this neurological outcome is particularly encouraging.

#### 4.1.1 Hemodynamic advantages of a physiological perfusion pathway

The innominate artery, as the first branch of the aortic arch, offers an anatomical position that allows ACP through this route to provide a hemodynamic environment more consistent with physiology. Compared to the circuitous path via the axillary artery, innominate artery ACP generates more stable perfusion pressure and more uniform cerebral blood flow distribution (8). Confirmed through computational fluid dynamics models that direct central perfusion can increase middle cerebral artery flow velocity by 25%−30%, significantly improving microcirculatory perfusion in brain tissue. The 10-mm large-bore artificial graft used in this study further optimizes hemodynamic properties (8). Fluid dynamics research indicates that compared to traditional 8-mm grafts, a 10-mm graft can reduce flow resistance by approximately 40% at the same flow rate. This enables the maintenance of an ACP flow rate of 8–10 ml/kg/min during HCA, ensuring cerebral oxygen saturation remains above 85% of baseline (9).

#### 4.1.2 Real-time monitoring and precise regulation

This technique achieved precise regulation of ACP flow through real-time monitoring of right radial artery pressure (maintained at 40–70 mmHg) combined with cerebral oxygen saturation monitoring (NIRS). Research by Olsson et al. showed that maintaining radial artery pressure above 50 mmHg can reduce stroke risk by 32 %. Simultaneously, NIRS monitoring ensured the stability of cerebral oxygen saturation, avoiding the issues of cerebral under-perfusion or over-perfusion possible with traditional methods (10).

### 4.2 Significant improvement in surgical efficiency and technical advantages

#### 4.2.1 Marked improvement in time efficiency

In this cohort, the mean innominate artery-to-graft anastomosis time was efficiently achieved in 5.0 ± 1.5. Although a formal statistical comparison was not performed, this duration is notably shorter than the times ranging from 15 to 20 that are commonly cited for surgical exposure and cannulation of the axillary artery (11). This efficiency contributes to a streamlined operative workflow and potentially reduces the ischemic preconditioning time. This time advantage holds significant clinical importance in ATAAD surgery, where time is of the essence. Rapid establishment of a reliable perfusion channel allowed for optimized control of CPB time (171.27 ± 43.65). Studies indicate that for every 30-min prolongation of CPB time, the risk of postoperative acute kidney injury increases by 23% (11). The relatively low incidence of acute kidney injury (23.40%) in this study partly reflects the positive impact of improved time efficiency.

#### 4.2.2 Optimized surgical field and operative convenience

All procedures were performed within the mediastinum, avoiding the surgical field interference associated with axillary dissection. This advantage is particularly evident during total arch replacement and frozen elephant trunk (FET) procedures. Operating within the central field allows the surgeon to focus more on key surgical steps, enhancing overall procedural precision and safety. Furthermore, the perfusion circuit is routed out from the superior aspect of the sternotomy incision does not interfere at all with the operative space around the aortic arch, a clear advantage over axillary artery cannulation (12).

### 4.3 Breakthrough in complication prevention

The most noteworthy finding of this study was the complete avoidance of specific complications associated with traditional cannulation techniques, which is highly significant in ATAAD surgery.

#### 4.3.1 Zero upper limb ischemia complications

Compared to the 2.3%−5.1% incidence of upper limb ischemia associated with traditional axillary artery cannulation (13), this technique completely eliminates this risk by preserving blood flow continuity to the right subclavian artery. The partial occlusion technique using side-biting clamps ensures continuous blood supply to the right upper limb while providing sufficient flow for CPB.

#### 4.3.2 Absence of brachial plexus injury

It avoids the 2%−5% risk of brachial plexus injury associated with axillary artery cannulation (14, 15). The axillary artery region has a complex distribution of the brachial plexus, and the risk of injury is significantly increased, especially in emergency settings and with specific patient positioning. This technique completely circumvents this risk, which is important for preserving postoperative quality of life.

#### 4.3.3 No retrograde aortic dissection

Compared to the 1.2%−2.8% risk of retrograde aortic dissection with femoral artery cannulation (16), the antegrade perfusion characteristic of this technique completely eliminates this potentially fatal complication. The high-velocity retrograde flow during femoral cannulation can exacerbate dissection propagation, whereas the physiological antegrade perfusion provided by this technique fundamentally avoids this risk.

### 4.4 Comparative advantages over existing techniques

Compared to axillary artery cannulation: although axillary artery cannulation is considered the gold standard for cerebral protection, it carries risks of brachial plexus injury, upper limb ischemic complications, and technical difficulties (17). Particularly in patients with poor vascular access or obesity, exposure of the axillary artery can be challenging and time-consuming. This technique avoids these drawbacks through central operative field access while providing superior cerebral perfusion.

Compared to femoral artery cannulation:

In distinction from femoral cannulation—which has declined in use due to the dangerous complication of retrograde aortic dissection extension—our method entirely circumvents this risk and also eliminates concerns of lower limb ischemia. The use of femoral artery cannulation in ATAAD patients has significantly decreased in recent years, primarily due to its associated risk of retrograde dissection.

Compared to other central cannulation techniques:

When evaluated against alternative central perfusion strategies, this technique demonstrates distinct advantages: compared to direct aortic cannulation, it avoids manipulation of the fragile dissected aorta; relative to subclavian artery cannulation, it affords improved surgical exposure and operative convenience.

### 4.5 Safety validation and technical reliability

The absence of any direct technique-related complications in this study confirms its safety and reliability. The innominate artery has sufficient wall thickness (typically 2–3 mm) and toughness to tolerate side-clamping and anastomosis. The 10-mm artificial graft matches well with the innominate artery, and the continuous exerting suture technique creates a hemodynamically excellent anastomosis.

Intraoperative and postoperative transesophageal echocardiography (TEE) monitoring showed stable flow through this perfusion pathway, with no signs of turbulence or thrombus formation. Postoperative CT angiography confirmed the patency of all anastomoses, with no stenosis or pseudoaneurysm formation.

### 4.6 Study limitations and future directions

As a single-center retrospective study, it has certain limitations. Firstly, the lack of randomized comparison with traditional cannulation methods means outcome comparisons rely mainly on historical control data. Secondly, although the sample size (94 cases) is relatively large for a single-center study, multi-center studies are needed to further validate the generalizability of the results. Furthermore, all surgeries were performed by an experienced aortic surgery team, and the learning curve factor needs to be considered during technology dissemination.

Future research should focus on the following directions: ① Conducting multi-center randomized controlled trials to directly compare the long-term outcomes of this technique with traditional axillary artery cannulation; ② Long-term follow-up to assess neurocognitive function outcomes, particularly neuropsychological evaluations at 6 months and 1 year postoperatively; ③ Further optimization of technical details, exploring more suitable artificial graft sizes and anastomosis techniques; ④ Further optimization of perfusion parameters through imaging and hemodynamic studies.

## 5 Conclusions

The modified perfusion strategy via anastomosis of the innominate artery with a 10-mm artificial vascular graft is a safe, effective, and technically reliable method for ATAAD surgery. While maintaining a mortality rate comparable to traditional methods, it significantly reduces the incidence of neurological dysfunction (10.64%), completely avoids specific complications like upper limb ischemia, and improves surgical efficiency by shortening anastomosis time. This technique is particularly suitable for preoperative critically ill ATAAD patients requiring complex arch procedures and holds significant value for clinical promotion and application prospects.

## Data Availability

The datasets presented in this study can be found in online repositories. The names of the repository/repositories and accession number(s) can be found in the article/supplementary material.

## References

[1] Isselbacher EM Preventza O Hamilton Black J 3rd Augoustides JG Beck AW Bolen MA . 2022 ACC/AHA guideline for the diagnosis and management of aortic disease: a report of the American Heart Association/American College of Cardiology joint committee on clinical practice guidelines. Circulation. (2022) 146:e334–482. doi: 10.1161/CIR.0000000000001097 36322642 PMC9876736

[2] Sakaguchi T Hiraoka A Totsugawa T Hayashida A Ryomoto M Sekiya N . Clinical impact of the repair technique for posterior mitral leaflet prolapse: resect or respect? J Card Surg. (2021) 36:971–7. doi: 10.1111/jocs.15312 33428267

[3] Rogers T Thourani VH. Transcatheter aortic valve replacement after mitral valve surgery: synergistic or incompatible? J Thorac Cardiovasc Surg. (2018) 155:66–7. doi: 10.1016/j.jtcvs.2017.09.071 29102215

[4] Botha P. Cardiac transplantation; for the benefit of the many, and the few. Ann Thorac Surg. (2023) 116:597. doi: 10.1016/j.athoracsur.2022.07.005 35863389

[5] Yamazoe M Mizuno A Nishi Y Niwa K. Arterial-pressure-based cardiac output analysis reveals the usefulness of pericardiocentesis. J Cardiothorac Vasc Anesth. (2015) 29:e26–8. doi: 10.1053/j.jvca.2014.11.004 25661640

[6] Gaudino M Audisio K Rahouma M Robinson NB Soletti GJ Cancelli G . Association between sternal wound complications and 10-year mortality following coronary artery bypass grafting. J Thorac Cardiovasc Surg. (2023) 166:532–9.e534. doi: 10.1016/j.jtcvs.2021.10.067 35063171

[7] Nappi F Spadaccio C Dreyfus J Attias D Acar C Bando K. Mitral endocarditis: a new management framework. J Thorac Cardiovasc Surg. (2018) 156:1486–95.e1484. doi: 10.1016/j.jtcvs.2018.03.159 29884490

[8] Treasure T Petrou M Rosendahl U Austin C Rega F Pirk J . Personalized external aortic root support: a review of the current status. Eur J Cardiothorac Surg. (2016) 50:400–4. doi: 10.1093/ejcts/ezw078 27032474

[9] Pacini D Leone A Di Marco L Marsilli D Sobaih F Turci S . Antegrade selective cerebral perfusion in thoracic aorta surgery: safety of moderate hypothermia. Eur J Cardiothorac Surg. (2007) 31:618–22. doi: 10.1016/j.ejcts.2006.12.032 17254793

[10] Terasaki T Takano T Fujii T Seto T Wada Y Ohtsu Y . Early and midterm results of transapical and right axillary artery cannulation for acute aortic dissection. J Cardiothorac Surg. (2015) 10:2. doi: 10.1186/s13019-014-0202-9 25573690 PMC4296545

[11] Karkouti K Wijeysundera DN Yau TM Callum JL Cheng DC Crowther M . Acute kidney injury after cardiac surgery: focus on modifiable risk factors. Circulation. (2009) 119:495–502. doi: 10.1161/CIRCULATIONAHA.108.786913 19153273

[12] Yamashita Y Sicouri S Dokollari A Rodriguez R Goldman SM Ramlawi B. Aortic versus axillary cannulation in acute type A aortic dissection repair: a meta-analysis. Asian Cardiovasc Thorac Ann. (2024) 32:234–43. doi: 10.1177/02184923241232008 38343086

[13] Wong DR Coselli JS Palmero L Bozinovski J Carter SA Murariu D . Axillary artery cannulation in surgery for acute or subacute ascending aortic dissections. Ann Thorac Surg. (2010) 90:731–7. doi: 10.1016/j.athoracsur.2010.04.059 20732486

[14] Zhang H Xie W Lu Y Pan T Zhou Q Xue Y . Double arterial cannulation versus right axillary artery cannulation for acute type A aortic dissection: a retrospective study. J Cardiothorac Surg. (2021) 16:326. doi: 10.1186/s13019-021-01714-5 34743732 PMC8574002

[15] Guo Q Dong D Zhou Q Huang S Qiao X Dang Z . The association between cardiovascular health and obstructive sleep apnea symptoms: findings from NHANES. Front Cardiovasc Med. (2024) 11:1466752. doi: 10.3389/fcvm.2024.1466752 39759500 PMC11695300

[16] Tiwari KK Murzi M Bevilacqua S Glauber M. Which cannulation (ascending aortic cannulation or peripheral arterial cannulation) is better for acute type A aortic dissection surgery? Interact Cardiovasc Thorac Surg. (2010) 10:797–802. doi: 10.1510/icvts.2009.230409 20154346

[17] Tong G Zhao S Wu J Sun Z Zhuang D Chen Z . Right axillary artery cannulation in acute type A aortic dissection with involvement of the right axillary artery. J Thorac Cardiovasc Surg. (2024) 168:50–9.e56. doi: 10.1016/j.jtcvs.2022.09.058 36464509

